# The interplay between social media use and problematic internet usage: Four behavioral patterns

**DOI:** 10.1016/j.heliyon.2023.e15745

**Published:** 2023-04-24

**Authors:** Khansa Chemnad, Maryam Aziz, Samir Brahim Belhaouari, Raian Ali

**Affiliations:** College of Science and Engineering, Hamad Bin Khalifa University, Qatar

**Keywords:** Problematic internet usage, Smartphone usage, Social media use, Cyber behaviour

## Abstract

**Objective:**

The study aims to identify typical interplay between the use of social media apps on smartphones and Problematic Internet Usage (PIU).

**Method:**

Our study utilizes data from a smartphone app that objectively monitors user usage, including the apps used and the start and finish times of each app session. This study included 334 participants who declared a need to be aware of their smartphone usage and control it. Problematic Internet Usage (PIU) was measured using the Problematic Internet Use Questionnaire-Short Form-6 (PIUQ-SF6). The total PIU score can range from 6 to 30, with a score above 15 indicating that a person is at risk of PIU. Time spent on Social Media (SM) apps of Facebook, WhatsApp, and Instagram, and whether people used each of these apps were studied along with the total PIU score. K-Prototype clustering was utilized for the analysis.

**Results:**

Four distinct clusters, typifying the relationship between social media use and PIU, were identified. All the individuals in Cluster 1 (*Light SM Use Cluster;* Cluster size = 270, 80.84% of total dataset) spent between 0 and 109.01 min on Instagram, between 0 and 69.84 min on Facebook, and between 0 and 86.42 min on WhatsApp and its median PIU score was 17. Those who were in cluster 2 (*Highly Visual SM Cluster; Cluster size* = 23, 6.89% of total dataset) all used Instagram, and each member spent between 110 and 307.63 min on Instagram daily. The cluster median PIU score and average daily usage of Instagram were respectively 20 and 159.66 min. Those who were in Cluster 3 (*Conversational SM Cluster; Cluster size* = 19, 5.69% of total dataset) all used WhatsApp, and spent between 76.68 and 225.22 min on WhatsApp daily. The cluster median PIU score and average time spent per day on WhatsApp were 20 and 132.65 min, respectively. Those who were in Cluster 4 (*Social Networking* Cluster; (Cluster size = 22, 6.59% of total dataset) all used Facebook, and each spent between 73.09 and 272.85 min daily on Facebook. The cluster median PIU score and average time spent per day on Facebook were 18 and 133.61 min respectively.

**Conclusion:**

The clusters indicate that those who use a particular social media app spend significantly less time on other social media apps. This indicates that problematic attachment to social media occurs primarily for one of three reasons: visual content and reels, conversations with peers, or surfing network content and news. This finding will help tailor interventions to fit each cluster, for example by strengthening interpersonal skills and resistance to peer pressure in the case of Cluster 3 and increasing impulse control in the case of Cluster 2.

## 1 Introduction

In recent years, extensive research has been conducted on the use of social media, owing to its exponential growth. As of July 2022, 4.70 billion people utilize social media, constituting 59% of the world population [1]. Most Facebook, Snapchat, and Instagram users report that they use these platforms daily [2]. On the one hand, technology has created new ways for people to establish connections and stay active and has promoted more reciprocal and continuous communication among people regardless of time or place. On the other hand, such innovative social communication methods have generated a new set of adverse effects. Online social networking has attracted increasing interest for its potential effect on well-being, with research exploring factors associated with both positive and negative mental health outcomes [3,4]. There are several reasons to use social media, including creating interpersonal connections, establishing a sense of identity, and learning about the world; however, excessive use can lead to problematic Internet use [5]. This can lead to an increased risk of developing mental health problems such as depression and anxiety, as well as sleep disorders, and decrease in physical activity [6,7]. Users have a multitude of social media platforms to choose from, and many are active on more than one platform [[8], [9], [10]]. The nature of social media is such that people often become addicted to it without realizing it [11].

Problematic Internet Usage (PIU) is defined in the literature as “the excessive use of the Internet that causes disturbances or harm to the individual” [12]. [13] described it as excessive Internet use that negatively affects one or more fundamental parts of an individual's life. Previous research has shown parallels between the symptoms of social media usage and Internet addiction [14]. Individuals who excessively use social media (SM) may exhibit Internet addiction symptoms [15]. People with PIU may have difficulty managing their Internet use and may have difficulties with their career or academic achievement, social relationships, and mental and physical issues [[16], [17], [18], [19]]. PIU can be difficult to identify and differentiate from normal use and may even go unrecognized by the individual experiencing it.

Social media (SM) platforms can be classified into different types based on their main features – primarily text-based such as Facebook [20], Highly visual social media networks (HVSM) such as Instagram and Snapchat, and instant messaging apps such as WhatsApp, and Telegram. HVSM networks are primarily used to share user-generated visual materials, such as photographs and short clips, and include an assortment of incorporated filters that allow digital manipulation of visuals before uploading. Most HVSM app users tend to be passive. There is evidence that HVSM platforms may cause persistent mental harm such as depression [21]. Owing to the pervasive nature of social media and peer pressure to use the Internet, it is easy to lose track of the time spent on social media platforms [11]. This has led to research on solutions that might assist individuals in self-regulating their use of digital media.

Instagram, Facebook, and WhatsApp are more prevalent than other social media platforms [22]. Facebook, WhatsApp, and Instagram are owned by the same company: Meta [23], and can be categorized as text and content-focused, conversational, and HVSM platforms, respectively. WhatsApp is a messaging app that allows individuals to send and receive text, voice, and multimedia messages and make audio and video calls [24]. According to previous studies, individuals spend more than half an hour every day using WhatsApp [25]. Facebook offers more features such as the ability to be part of groups, follow pages of interest, and subscribe to news sites, as well as the ability to sell and buy things, share and read posts (e.g., videos, photographs, text, or combinations of these), and update status [26]. Instagram is a free photo and video-sharing application for iOS and Android devices. Individuals may use this service to post photographs or videos and share them with their followers or a small group of friends. Additionally, they may browse, comment on, or like Instagram posts shared by their friends [27]. As of December 2021, Instagram has reached a new milestone of 2 billion active users globally [28]. Since Instagram's adoption is rapidly increasing, research on this topic is still in its infancy.

### 1.1 Literature review

Social media platforms have become an integral part of modern life, allowing people to communicate with one another in various ways. From instant messaging apps such as WeChat, Messenger, and WhatsApp, to image-based platforms such as Instagram, and even professional networking sites such as LinkedIn, social media takes on many different forms. Each type of social media application caters to different social needs and fosters different user behaviors [29]. This raises the question whether users who use social media excessively, use some or all types of social media apps and whether we can distinguish among different socialisation needs, accordingly. Attachment type influences the extent to which social media usage affects users' mental health and well-being [4]. People with low avoidant attachment and high anxiety attachment are more prone to problematic social media use, and low psychological well-being. According to Ref. [30], Instagram users value self-promotion and self-identity more than connectivity and other motives, such as supporting their identity, acquiring knowledge about others, documenting life events, and displaying their creativity, such as photographs. Recent evidence suggests that frequent Instagram usage is related to problematic smartphone use [31]. According to a survey conducted in the United Kingdom by the Royal Society for Public Health [32], Instagram was the most harmful social media site in terms of its effect on adolescents' well-being. Users' interactions can also classify social media apps as actively social, passively social, and content focused. Active use of social media sites includes any activity that allows direct exchange with others, such as direct communication, posting, sharing private links, or direct messaging. In contrast, passive use involves monitoring other people's lives without direct engagement, such as scrolling through profiles, pictures, videos, or updates [33]. Research has also demonstrated that passive social apps foster social comparison and jealousy, which have a detrimental impact on subjective well-being [33]. According to Ref. [34], smartphone use disorder was found to be higher for those with Instagram Use Disorder, and WhatsApp Use Disorder than those with Facebook Use Disorder, and Snapchat Use Disorder.

The predictors of excessive use of each of these social media platforms are different in the literature. For example, low openness and low conscientiousness were found to be predictors of excessive Facebook use [35], whereas high neuroticism and low agreeableness were found to be predictors of excessive Instagram use [36]. People with high extraversion levels use messaging applications more often and for longer periods, make more outgoing phone calls, and send and receive more text messages than those with low extraversion levels do. Additionally, high anxiety, low self-esteem, and fear of missing out (FOMO) are associated with excessive use of WhatsApp [37]. Problematic smartphone use in relation to problematic WhatsApp use, problematic Facebook use, and problematic Instagram use was studied in Ref. [38]. The research demonstrated that, whereas problematic Facebook and Instagram usage seem to be separate occurrences, problematic smartphone and WhatsApp use were inextricably linked [38]. While some studies have explored PIU related to social media apps, they were limited by self-reported data and the fact that they did not consider the possibility of people simultaneously developing multiple problems at the same time [39,40]. Because the predictors and functionalities of apps are different, in this paper we hypothesize that people who exhibit excessive use of one platform may not exhibit excessive use of other platforms at the same time.

The fear of missing out (FoMO) is a prevalent issue among social media users [41]. However, the expression of FoMO may vary among social media app users. FoMO is a phenomenon commonly described as a persistent sense of anxiety or worry that other people have enjoyable experiences without their participation and is often marked by a strong need to stay updated on others' activities [42]. Lack of self-control is a risk factor for mental health, and social media addiction and fear of missing out (FoMO) further raise this risk [43]. For example, users of WeChat, Messenger, and WhatsApp, which are primarily messaging apps, demonstrate distinct behavioral patterns compared with those of image-based social networking platforms such as Instagram. The fear of missing out on WeChat, Messenger, and WhatsApp may arise from fear of ostracism, which is the fear of exclusion, neglect, or exclusion by others. This type of FOMO is motivated by the desire for social belonging and validation, and users of these applications may experience anxiety or stress if they are left out of group chat or discussion. However, Instagram users may experience FoMO from the need to know what is going on or who has liked a post. This type of FoMO focuses on social validation, rather than on developing personal relationships. Instagram users may feel pressured to constantly check their feed to stay up-to-date with the latest trends and to ensure that they are not missing out on any important events or updates. Rozgonjuk et al. [44] explored the relationship between FoMO and social media use disorders that negatively affect daily life, revealing that, while all platforms demonstrated a mediating effect except for Snapchat. Because FoMO arising from the use of different social media apps are different, in this paper we hypothesize that individuals may not exhibit addiction to all of the social media apps at the same time.

The theoretical framework of the present study was based on three major explanatory models of problematic use of social media: the Uses and Gratification Theory 2.0 [45], the Compensatory Internet Use Theory [46], and the I-PACE (Interaction of Person-Affect-Cognition-Execution) model [47]. The fundamental concept of the Uses and Gratification (U&G) theory is that people would actively seek specific media that fulfills their needs and ultimately satisfies them [48]. Furthermore, the revised Uses and Gratification (U&G 2.0) Theory 2.0 demonstrates how technology actively impacts human–media interactions [45]. People have certain preferences that influence their choices of media. These preferences may include the design of the platform, the content, and the features offered. Therefore, people may have different motives for using specific platforms. Additionally, people may be drawn to certain platforms based on their social connections [49]. This suggests that the relationships between people and technology are complex and highly individualized. Moreover, these preferences can change over time as technology advances. Another theory that has been used in the literature to explain excessive Internet use is the Compensatory Internet Use Theory (CIUT) [46], which proposes that individuals may turn to the Internet, including social media platforms, as a way of compensating for unmet needs in their offline lives. In the context of excessive social media use, CIUT suggests that individuals use these platforms to compensate for specific deficiencies or challenges in their offline lives. This suggests that unmet needs may differ depending on the individual. Hence, there is a possibility that they end up utilizing one social media app more than the other. CIUT has been demonstrated to be effective in conceptualizing PIU and excessive social media use [50,51]. The I-PACE (Interaction of Person-Affect-Cognition-Execution) model is another theoretical paradigm that attempts to explain the emergence and maintenance of addictive behaviors, such as excessive social media usage [52]. According to the model, addictive behaviors result from a combination of individual risk factors, emotional and cognitive reactions to internet usage, and the capacity to execute and manage internet-related behaviors, which may result in either positive gratification or excessive use.

Platforms such as Facebook and Twitter are often referred to as “social media”. However, these platforms differ significantly in terms of their structure and functionality, and the ways in which users use them [53]. Although social media usage has been extensively studied, few have objectively investigated the relationship between PIU and social media usage [54]. Social Media usage and addiction have mainly been studied using offline methods of data collection such as surveys and interviews, which can be biased by recall and lack ecological validity [55]. Collectively, these studies indicate that the way users spend time on different apps needs to be studied to design more effective intervention programs. Screen time has primarily been used as a measure to study PIU; however, screen time alone is not sufficient to study PIU [56]. As different social media platforms incite different kinds of behavior, interventions need to be designed separately as well. Hence, using screen time as a holistic measure is insufficient to study PIU and design interventions. More evidence-based intervention programs are required to prevent PIU [57]. We will examine how people spend their time on various social media sites, such as HVSM platforms (e.g., Instagram), networking platforms (e.g., Facebook), and messaging services (e.g., WhatsApp). By doing so, we are able to go beyond research of single social media sites and instead concentrate on how people utilize various social media platforms. This method to evaluating social media usage is more likely to represent an individual's social media ecology. Social media ecologies classify the many channels and modes of communication that people utilize depending on their interpersonal ties and the perceived features and functions of various platforms [58]. By evaluating the interaction across numerous platforms, we may get closer to gauging the social media ecologies that people develop for their everyday needs, whether they use one, some or all the platforms excessively.

### 1.2 Research questions

In this paper, we collect smartphone usage data objectively through a designated software and study the typical interplays between the usage of three social media platforms: Facebook, WhatsApp, and Instagram, and Problematic Internet Usage through clustering. These apps are representative of three different categories of Social Media: text and content-focused, conversational, and HVSM platforms. The clusters identified will help design and facilitate intervention programs for users depending on their pattern of social media app usage to help reduce PIU.

While prior research has investigated the excessive use of individual social media platforms [59], there is a lack of research on addiction across multiple platforms and the possibility of simultaneously developing addiction on them. Additionally, previous studies have treated social media as a homogeneous entity or examined each platform individually, without considering the possibility of grouping users based on their usage patterns across multiple social media platforms. Thus, this study aims to investigate whether distinct user groups can be identified based on their use of distinct social media platforms.

RQ1 Can social media usage patterns on Instagram, WhatsApp, and Facebook, and the time spent on each platform, be used to identify user groups based on their levels of problematic Internet use (PIU)?

Platform swinging, the habit of using multiple social media platforms and constantly switching between them, is common and prevalent across all ages [60]. However, literature on social media addiction has mostly focused on individual platforms, ignoring the potential co-occurrence of addiction on numerous platforms. Given that social media use is becoming more complicated and diverse, and that users often interact with different platforms simultaneously, it becomes necessary to comprehend the implications of the probable co-occurrence of addiction on the spectrum of social media types. Additionally, understanding the complexity of social media addiction and its multiple platform use could aid in the development of more specific prevention and intervention programs. Therefore, this study aims to investigate whether individuals can develop excessive use of multiple social media apps, considering that these platforms differ in functionality and the psychological needs they may fulfil.

RQ2 Are individuals who excessively use one social media platform also prone to excessive use on other platforms or is problematic Internet usage specific to certain platforms?

## 2 Methodology

### 2.1 Dataset

The dataset for this study was collected through the company owning and managing an Android third-party app that helps users track their smartphone use. The data collected from the app include the name of each app used and the start and end times of each use session. Demographic data of age, gender, and country were also collected as part of the user registration to the app. The privacy statement of the app specifies that acquired data can be shared with academic partners for research purposes. The users were still asked explicitly to consent to the collection and sharing of their data anonymously. Users were given a premium version of the app as a token of appreciation for those who accepted it. The request to participate in this study was issued to users who started the registration process upon installation of the app. The data were collected between October 2020 and April 2021. The study was reviewed and approved by the Institutional Review Board (IRB) of the first author.

### 2.2 Data preparation

Python 3.0 was used to pre-process the data, and to perform the statistical analysis for the whole study [61]. The data preprocessing steps included removing duplications in the data records, calculating app usage time, and checking for apparent anomalies. We also unified the dates format and language as users came from different countries. Since the app is available only on Google Play, all participants in the study were Android users. The app tracks all the apps that a user uses and each session's start and end timestamps.

Although 602 participants consented to data collection, only 334 met the inclusion criteria. Only users who had used either one of the three apps of Instagram, WhatsApp, or Facebook were included. We considered a one-week usage data, and only included users who had at least five days of smartphone usage. Some users uninstalled the app in the first five days and were excluded. Earlier research found that a minimum of five days is necessary to accurately depict weekly usage habits [62]. The present study did not employ specific selection criteria, except for the requirement of participants to complete at least one week of usage. For the purposes of this study, we did not consider age, gender, or cultural background as factors that could potentially affect the outcomes. This decision was based on the mixed results found in the literature regarding the roles of age [63,64], gender [63,[65], [66], [67]], and culture [68,69] in PIU. Although our sample was naturally balanced in terms of gender, some participants did not report their gender and this would have limited our sample size. Furthermore, owing to the sample size, it was not possible to consider other variables that may have influenced the results.

### 2.3 Measures

#### 2.3.1 Social media app average use

Three continuous variables (Facebook Usage, WhatsApp Usage, and Instagram Usage) were computed and recorded to denote the average daily use of each of the apps by the user. The average was computed using the total time spent in minutes on each app over the week.

#### 2.3.2 Has social media app use?

Three binary variables (Has Facebook Use? Has WhatsApp Use? Has Instagram Use?) were recorded to indicate whether the user had used any of the apps.

#### 2.3.3 Problematic internet use (PIU)

PIU was measured using the 6-item Problematic Internet Use Questionnaire Short Form (PIUQ–SF–6), a self-reporting scale [70]. PIUQ–SF–6 measures three key aspects of problematic Internet usage: preoccupation, neglect, and control disorder. A previous study has shown that shorter scales with fewer questions are more accurate for evaluating problematic Internet usage with survey fatigue's impact on research findings [70]. The PIUQ–SF–6 scores can vary from 6 to 30, with higher values suggesting greater PIU. The scale items are assessed on a 5-point Likert scale (from “never” to “always/almost always”). According to Ref. [70], a PIU total score greater than 15 indicates that a person is at risk of Problematic Internet Use. Internal consistency was assessed using both McDonald's Omega and Cronbach's alpha and was found to be 0.70 in both cases indicating acceptable reliability [71,72].

### 2.4 Data analysis

Python 3.0 was used to conduct the analysis for this research [61]. Unsupervised clustering analysis was applied to determine the interplay between different social media app usage regarding PIU. The divide and recursive merge approach was used to identify the clusters. This approach generates and recursively merges the maximum number of clusters based on hypothesis testing to obtain significantly different clusters [73]. Categorical variables of Has WhatsApp? Has Facebook? and Has Instagram? and continuous variables of Total PIU Score, Instagram average time, Facebook average time, and WhatsApp average time were utilized in the clustering analysis. The K-Prototype clustering approach was chosen due to having both numerical and categorical features in the data. It uses Euclidean distance to measure the distance between numerical variables, and it uses the Hamming distance (number of matching categories) to evaluate the distance between categorical features. Correlation coefficients between the clustering variables were examined.

**Fig. 1:**
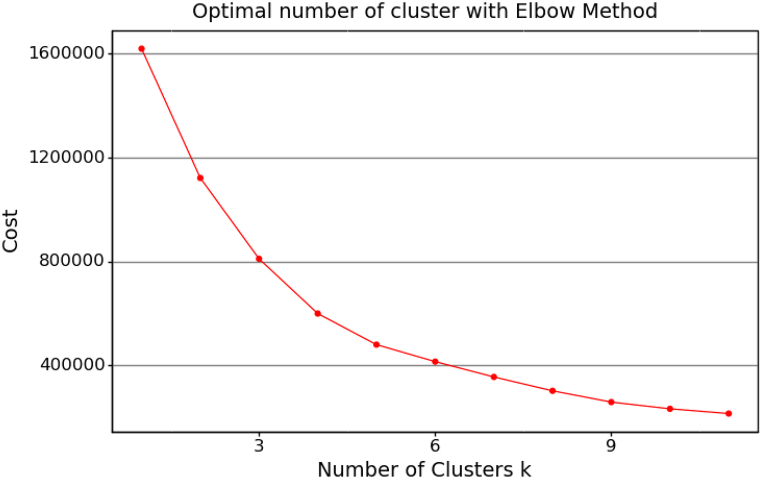
Elbow plot with the maximum number of clusters.

The elbow method was used to determine the maximum number of clusters in the division phase. The elbow method runs the K-prototype algorithm for each cluster, and calculates the cost for each cluster size. To mitigate the instability of the K-prototype method, we ran the algorithm a few times with various clusters ranging from one to 12. The sum of all dissimilarities between the clusters was averaged and plotted to determine the cost. The elbow plot from the division phase is shown in Fig. 1. Considering that the curve follows an approximately linear route after it, the maximum number of clusters was taken as four.

A *t*-test analysis was used to merge the clusters in the recursive merging phase. We used the t-statistic instead of the p-value to determine whether a merge would occur or not. The p-value helps to identify whether the cluster centroids are significantly different but does not provide enough evidence on whether the centroids are similar. The clusters' centroids were taken as the mean for numerical attributes and mode for categorical attributes of the clusters to obtain the best representation of the cluster's center.

**Fig. 2:**
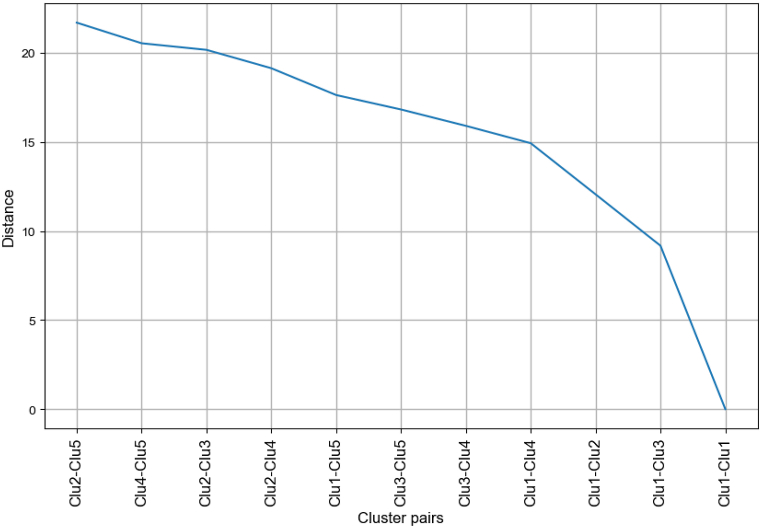
Distance between each cluster pair.

Fig. 2 shows the distance between the centroids of the clusters, where two possible threshold values can be observed for clustering. Taking a higher threshold of approximately 15 results in the clusters merging into one; hence we chose the second threshold value of 10. Clusters with distances below the threshold were merged. After merging, four final clusters were obtained.

## 3 Results

### 3.1 Descriptive statistics

The sample consisted of 190 females (56.89%), and 122 males (36.53%), and the rest chose not to answer their gender. All participants came from the following ten countries: Australia, Brazil, Canada, Germany, France, India, Netherlands, Sweden, the United Kingdom, and the United States. As for the age of the participants, 41.32% were emerging adults (18–24), and 3% chose not to answer.

A total of 334 participants were part of the study. Table 1 summarizes the descriptive statistics of the variables used in the clustering analysis.

**Table 1:** Descriptive statistics of variables.

Categorical Variables			N (334)	%
Has Facebook Usage? (YES)			170	50.90
Has Instagram Usage? (YES)			238	71.26
Has WhatsApp Usage? (YES)			240	71.86
**Numerical Variables**	**Mean**	**Std. dev.**	**Skewness**	**Kurtosis**
Facebook Usage (Minutes)	35.26	46.33	2.46	6.98
Instagram Usage (Minutes)	46.68	49.28	1.95	5.41
WhatsApp Usage (Minutes)	32.24	37.97	2.24	6.05
Total PIU Score	18.23	4.52	0.22	−0.25

### 3.2 Analysis

**Fig. 3:**
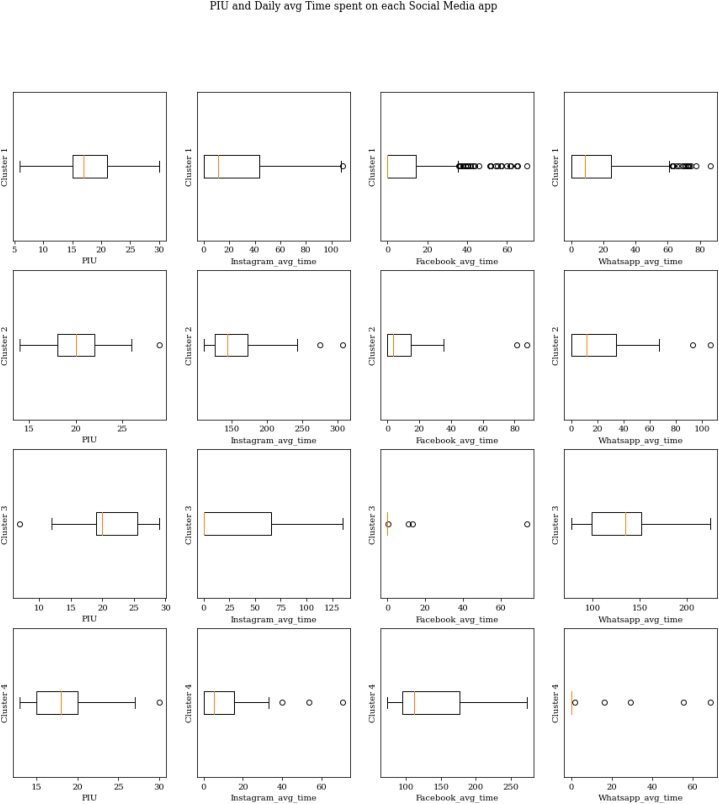
Box plot of the Cluster Variables with PIU and Average Time on Each App.

From the analysis, four clusters were discovered: Cluster 1 (Cluster size = 270, 80.84% of the total dataset), Cluster 2 (Cluster size = 23, 6.89% of total dataset), Cluster 3 (Cluster size = 19, 5.69% of the total dataset), and Cluster 4 (Cluster size = 22, 6.59% of the total dataset). Fig. 3 shows the boxplots of the features of the final clusters.

#### 3.2.1 Cluster 1 (Light Social Media Cluster)

Among the 270 individuals in cluster 1, 191 use Instagram (70.74%), 130 use Facebook (48.15%), and 199 use WhatsApp (73.70%). Fig. 3 shows that 25% of the individuals in this cluster had a PIU score of 21 or higher, with PIU scores ranging from 6 to 30. Around 50% of the people in this cluster had a PIU score between 15 and 21. The median PIU in this cluster was 17, indicating that people were at a risk of PIU.

**Fig. 4:**
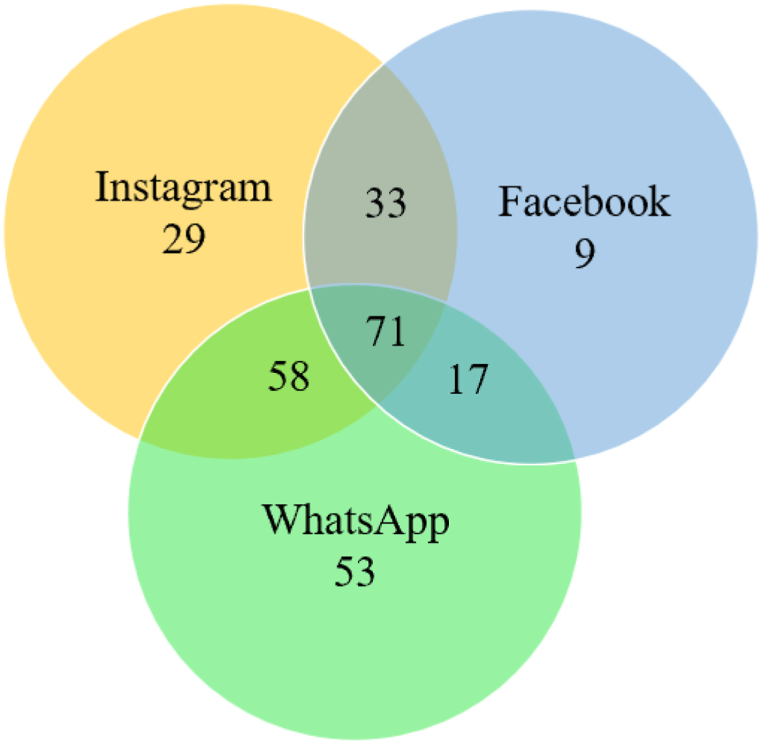
Individuals using the different apps in Cluster 1 (Cluster size:270; count).

The average time spent on each app and average PIU were calculated by computing the cluster means. All people in this cluster spent less than an average of 25 min per day on either of the three apps. Cluster 1's daily use of Instagram ranged between 0 min and 109.01 min, daily use of Facebook ranged between 0 and 69.84 min, and daily use of WhatsApp ranged between 0 and 86.42 min. The average daily use was 24.39 min on Instagram, 9.77 min on Facebook, and 16.63 min on WhatsApp. The mean PIU score for this cluster was 17.87. The Venn diagram in Fig. 4 indicates the number of individuals using each app in Cluster 1.

#### 3.2.2 Cluster 2 (Highly Visual Social Media (HVSM) Cluster)

Among the 23 people in this cluster, 23 (100%) use Instagram, 14 use Facebook (60.87%), and 17 use WhatsApp (73.91%). 25% of the individuals in this cluster had a PIU score of 22 or higher, with PIU scores in this cluster ranging from 14 to 29.50% of the people in this cluster had a PIU score between 18 and 22. The median PIU in this cluster was 20, indicating that people were at a risk of PIU.

**Fig. 5:**
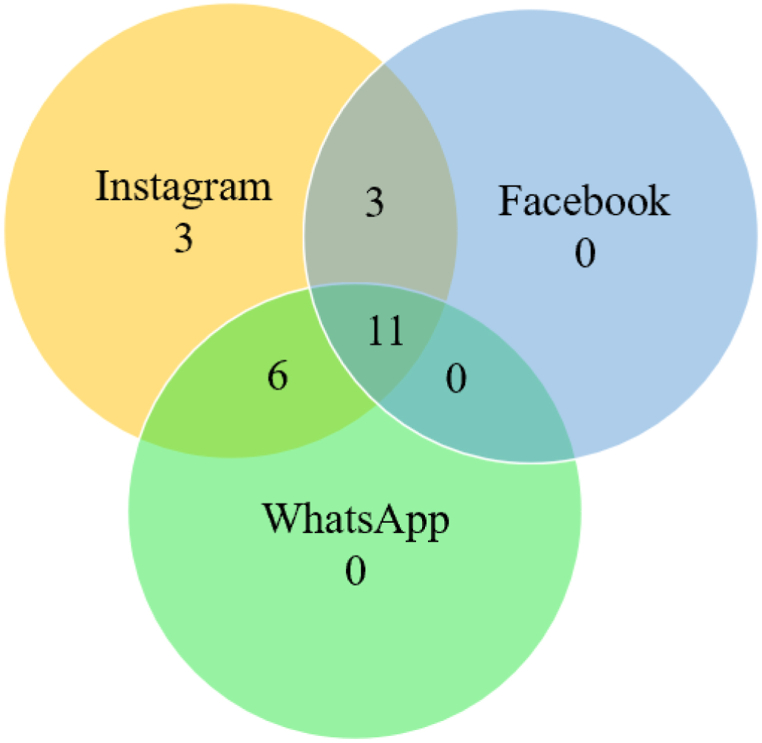
Individuals using the different apps in Cluster 2 (Cluster size:23; count).

The minimum time spent on each app was calculated by computing the cluster minimum. The average time spent on each app and average PIU were calculated by computing the cluster means. Individuals in this cluster spent a minimum of 110 min on Instagram daily. Cluster 2's daily use of Instagram ranged between 110.60 min and 307.63 min, daily use of Facebook ranged between 0 and 87.57 min, and daily use of WhatsApp ranged between 0 and 106.22 min. Cluster 2 average daily use was 159.66 min on Instagram, 13.85 min on Facebook, and 24.11 min on WhatsApp. The mean PIU score was 20.35. The Venn diagram in Fig. 5 indicates the number of individuals using each app in Cluster 2.

#### 3.2.3 Cluster 3 (Conversational SM Cluster)

Among the 19 individuals in Cluster 3, 19 use WhatsApp (100%), 8 people use Instagram (42.11%), and 4 people used Facebook (21.05%). 25% of the individuals in this cluster had a PIU score of 25.50 or higher, with PIU scores in this cluster ranging from 7 to 29.50% of the people in this cluster had a PIU score between 19 and 25.50. The median PIU in this cluster was 20 indicating that the people were at risk of PIU.

**Fig. 6:**
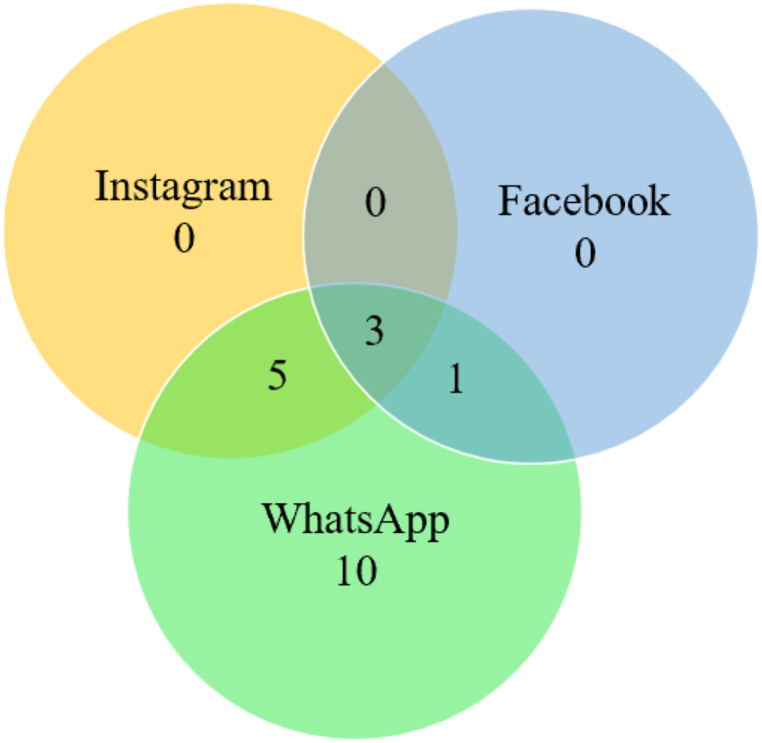
Individuals using the different apps in Cluster 3 (Cluster size: 19; count).

The minimum time spent on each app was further calculated by computing the cluster minimum. The average time spent on each of the apps was calculated by computing the cluster means. Individuals in this cluster spent a minimum of 76.68 min per day on WhatsApp. Cluster 3's daily use of Instagram ranged between 0 min and 135.26 min, daily use of Facebook ranged between 0 and 73.61 min, and daily use of WhatsApp ranged between 76.68 and 225.22 min. Cluster 3 average daily use was 132.65 min on WhatsApp, 5.18 min on Facebook, and 29.09 min on Instagram. The mean PIU score was 20.95. The Venn diagram in Fig. 6 indicates the number of individuals using each app in Cluster 3.

#### 3.2.4 Cluster 4 (Social Networking Cluster)

Among the 22 individuals in Cluster 4, 22 use Facebook (100%), 16 use Instagram (72.73%), and 5 use WhatsApp (22.73%). 25% of the individuals in this cluster had a PIU score of 20 or higher, with PIU scores in this cluster ranging from 13 to 30.50% of the people in this cluster had a PIU score between 15 and 20. The median PIU in this cluster was 18 indicating that people were at a risk of PIU.

**Fig. 7:**
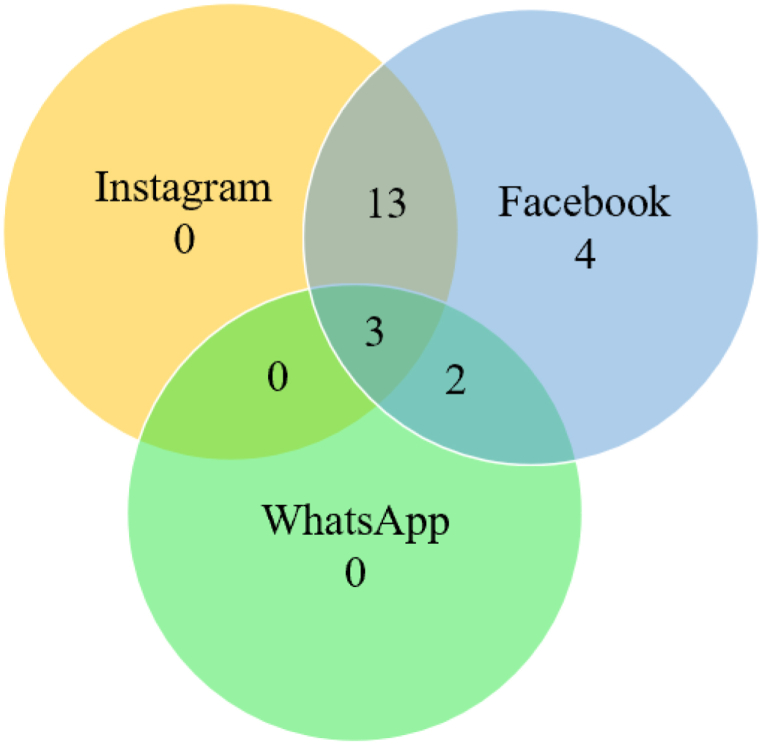
Individuals using the different apps in Cluster 4 (Cluster size: 22; count).

The minimum time spent on each app was further calculated by computing the cluster minimum. The average time spent on each of the apps was calculated by computing the cluster means. The 22 people in this cluster spent a minimum of 73.09 min per day on Facebook. Cluster 4's daily use of Instagram ranged between 0 min and 71.04 min, daily use of Facebook ranged between 73.09 and 272.85 min, and daily use of WhatsApp ranged between 0 and 68.60 min. Cluster 4 average daily use was 133.61 min on Facebook, 13.5 min on Instagram, and 7.8 min on WhatsApp. The mean PIU score was 18.09. The Venn diagram in Fig. 7 indicates the number of individuals using each of the apps in Cluster 4.

## 4 Discussion

The main objective of this study was to understand the typical interplays between social media usage and PIU. The analysis revealed four unique clusters that correspond to various patterns of social media usage and PIU: light social media users, conversational SM cluster, social networking SM cluster, and highly visual SM cluster. Each cluster has distinct qualities and behaviors associated with the time people spend on each social media app and their PIU. According to recent research, one of the negative consequences of social media is the possibility of addiction and its detrimental influence on personal and professional environments [74]. The results of K-prototype Clustering revealed that people who spent more time on WhatsApp had higher PIU than those who spent more time on Instagram or Facebook. Descriptive analysis showed that people who use each of the apps of Facebook, Instagram, and WhatsApp spent an average of 35.26 min per day on Facebook, 46.68 min per day on Instagram, and 32.24 min on WhatsApp, respectively.

The light social media cluster exhibited relatively low PIU among the rest of the clusters indicating the importance of measuring the use of different types of social media platforms when studying PIU. The behavior of individuals in this cluster may be explained by the Uses and Grats 2.0 theory, where individuals use each platform in a balanced manner to satisfy a range of needs, such as communication, entertainment, and social connections [45]. Their use of these apps may not be driven by excessive or addictive behavior but rather by a desire to meet their personal and social needs in a positive and fulfilling manner.

Users in the conversational cluster exhibited higher PIU than the Highly Visual Social Media and Social Networking clusters. The conversational cluster comprised of those who spent significantly more time using WhatsApp. These results seem consistent with those of [75], who showed that Smartphone Use Disorder was linked to WhatsApp Use Disorder more than Facebook Use Disorder. According to Ref. [44], WhatsApp was more associated with Problematic Smartphone use, and problematic Facebook and Instagram Use were reported to be a distinct phenomenon. A possible explanation for the behavior of individuals in this cluster may be based on the CIUT that individuals may use WhatsApp excessively to compensate for a lack of social support or connection in their offline lives [76]. For instance, an individual could use WhatsApp to communicate with friends or family members who are not nearby if they are going through a tough moment or feel socially isolated [76]. Although this may be a useful way to stay connected, excessive usage of the platform may interfere with other critical aspects of one's life, such as employment or personal relationships [77,78]. Another possible explanation may be based on the I-PACE model, where those who are susceptible to addictive characteristics, such as impulsivity, may be more likely to develop addiction to WhatsApp [40,47]. Affective and cognitive reactions to WhatsApp usage, such as feelings of social connectivity or satisfaction from receiving messages, may reinforce this behavior. Therefore, difficulties in controlling and monitoring WhatsApp usage may lead to the development and maintenance of its excessive use.

People in the Social Networking cluster had less PIU than those in the Highly Visual Social Media and conversational clusters did. The social networking cluster consisted of those who spent significantly more time on Facebook. These results further support the idea of [79], who studied the difference in problematic usage between Instagram, and Facebook using the Bergen Social Media Addiction Scale [80], and reported that Instagram users had substantially more problematic usage behavior than Facebook users. A possible explanation for the behavior of people in this cluster may be based on the I-PACE model [52]. Factors such as personality traits may predispose individuals to excessive Facebook use. For instance, those who are more outgoing or have a greater desire for social connections may be more inclined to indulge in excessive Facebook networking [81]. Recent research has reported that individual differences influence how people interact with Facebook [82]. Individuals often shift between active social, active non-social, and passive usage, at least on Facebook, and heavy users may participate in all three within the same session [83]. A multitude of subjective well-being indicators have been connected to using Facebook to enhance social capital, providing evidence that active usage of Facebook is associated with higher subjective well-being [84], which could have resulted in lower PIU in this group.

The Highly Visual Social Media cluster exhibited a relatively higher PIU than the Social Networking cluster. This cluster consisted of those individuals who spent significantly higher time on Instagram compared to Facebook or WhatsApp. These results align with previous findings, which found that Instagram users had substantially more problematic usage behavior than Facebook users [79]. The behavior of people in this cluster may be explained based on CUIT, where individuals may use Instagram excessively to compensate for poor self-esteem or a lack of feeling of self-worth [[85], [86], [87]]. For example, someone who is self-conscious of their looks or capabilities may turn to Instagram for validation or affirmation in the form of likes, comments, or followers. Although this may offer a short boost to self-esteem, excessive use of the platform may eventually increase feelings of inadequacy and promote a cycle of seeking external validation. The need to satisfy social demands on Instagram regularly may lead Instagram users to create a feeling of belonging to the site and, as a result, end up getting attached to it [88]. Another possible explanation may be based on I-PACE, where individuals who are predisposed to addictive behaviors, such as low self-esteem, may be more likely to develop excessive use of Instagram [47]. The satisfaction obtained from obtaining likes or favorable comments on posts may reinforce this behavior. Individuals may also struggle to regulate their emotional reactions to Instagram usage, leading to even more problematic behaviors.

An interesting finding from the analysis is that those who were highly accustomed to a particular social media app did not spend excessive time on other types of apps. It can be said that when people are attached to a particular social media app, they become obsessed with it. This can be explained based on the earlier observations of [89], who reported that a person who is addicted becomes “fixated” on a string of compulsive habits, losing their flexibility and susceptibility to novelty. This fixation culminates in a loss of interest in all other activities. Whether people belonged to the Light SM, Highly Visual Social Media, Conversational, or Social Networking clusters as per the PIU score, they were still at risk of PIU since all of their average scores were above 15 [70]. Comparing and understanding how various social media platforms affect users might assist in identifying which features of social media usage are positive and which have a negative influence on users across platforms [83].

### 4.1 Theoretical contributions

Our study has several strengths. First, our research on excessive social media use differs from previous studies, which relied mostly on linear models such as structural equation Modelling, and multiple linear regression [40]. Instead, we used a K-prototype clustering method.

Second, while most studies relied on self-reporting [40], we have objectively observed data, which is a strength of this study. The self-reported technology usage time is less reliable than objective methods [55,90]. Furthermore, the literature has studied each app individually or on social media as a whole. To the best of our knowledge, this is the first study to examine the interaction between the usage time of different apps regarding PIU.

Third, we used clustering, which is a powerful tool for grouping individuals based on shared characteristics and traits. This approach is often used in the context of healthcare and offers several advantages when designing interventions, including personalization, targeting, and efficiency [91]. Practitioners may create interventions that are more likely to be successful and well-received if they identify subgroups with comparable traits or needs. Using clustering, practitioners may target interventions to individuals who are most likely to benefit from them aiding in conserving time, effort, and streamlining interventions [92].

Fourth, the findings suggest that social media use may not be a uniform construct and that different apps may have unique effects on problematic internet usage. Identical addiction scores may contribute to vastly different experiences and subsequently have varying impacts on well-being. The results further demonstrate that not all addiction to social media apps is the same. Our results show that one of the three factors—conversation with peers, browsing network material and news, or engaging with visual content and reels—is the primary cause of problematic social media use.

Finally, through the light social media cluster, the research indicates that users who were not high in PIU seemed to have balanced use of social media and perhaps view multiple social media platforms as part of a cohesive unit that supports each other in fulfilling different needs. This is done through various settings and features offered by the platforms. Furthermore, other clusters suggest that individuals who develop addiction to one platform tend not to spend much time on other social media platforms. This aligns with current efforts to build theories that can be applied to multiple platforms, freeing researchers from the need to study each new platform with identical methods and theories, resulting in similar outcomes.

### 4.2 Practical implications

The results of our study have several practical implications. It is important for individuals to examine how they use social media and find any unmet needs that may be behind their excessive use. Individuals may work towards building healthy habits surrounding their social media usage and addressing any unmet needs in their life by identifying their motives for using the network. People can develop self-regulation skills such as establishing use limits, monitoring their emotions while using social media, and practicing mindfulness. This may assist people in better controlling their social media usage and reducing the negative impact of excessive use.

Our findings shall help tailor interventions for each of the clusters. For those addicted to visual content and reels, interventions that focus on increasing impulse control could be beneficial. For those whose addiction stems from conversations with peers and browsing network material, research has shown that interventions that help strengthen interpersonal skills, and being taught to resist peer pressure may be effective [93]. The plans for intervention may also benefit from the assessment of inter-individual factors such as peer relationships, family relationships, and social support for each cluster [94]. Positive Psychology Intervention is an effective treatment for PIU, particularly for reducing Internet usage and enhancing the quality of social connections [95]. Positive psychology intervention refers to individual/group-based therapy strategies that boost positive emotions and may improve an individual's social interactions.

From an industry perspective, social media platforms and Internet service providers are responsible for ensuring that their users safely and responsibly utilize their products. These firms may design measures to mitigate the negative consequences of social media and Internet usage by identifying elements that lead to problematic Internet use. For example, they may include features that restrict the amount of time users spend on their platforms or they may provide tools to assist users in developing healthy technological habits. Overall, promoting healthy social media habits and addressing underlying issues that drive excessive use can help individuals and organizations mitigate the negative impacts of social media on well-being.

### 4.3 Limitations

Our study has a few limitations. Only three of the platforms were considered owing to the limited number of users who used other social media apps in our sample. However, insights from studies of one platform may be interpreted in the context of other platforms that have the same features and functions by concentrating on high-level features that transcend individual technology when analyzing social media usage and results [96]. Our choice considered each of the different types of social media – Social Networking, Highly Visual, and Conversational. Although objectively collected data were used in our study, a limitation is that people in the dataset could be using multiple devices to access social media platforms. Our results shall be interpreted with caution as we only studied mobile social media usage. Concerning the relationship between social media use and PIU, research has shown that in addition to the time spent on social media, the motivation and intention of using these apps need to be considered while studying their relation to PIU [97]. Social Media platforms are continually extending and changing their features. Certain features that may affect time spent on the platform may not have been captured during the data collection process. For example, Instagram introduced a Watch Together feature – a feature where friends could tune into IGTV videos and reels in real-time over video chat [98] in November 2020, which was during the period of data collection. Although we initially wanted to observe how much time was spent on other apps such as utility apps, and communication apps in addition to Social Media apps, the sample size was a limiting factor that did not allow for the inclusion of other apps within this study. Additionally, although social media apps and social networking apps have been interchangeably used in literature, they are not the same [99]. Therefore, the survey questions consisted of the PIUQ-SF 6 scale instead of the six-item Bergen Social Media Addiction Scale (BSMAS) [80]. The BSMAS was developed based on apps such as Facebook, Instagram, Twitter, and the like. WhatsApp, which is prominently messaging app, was not part of it. The PIUQ-SF 6 scale is a reliable and widely used measure of Internet addiction that allowed us to focus on assessing excessive use of any of the app, regardless of whether or not they were officially categorized as social media. This decision provided a more accurate representation of PIU in the sample population, given our limitations on app inclusion.

### 4.4 Future work

Future research on social media and addiction should include more social media platforms that exhibit other characteristics and attract different populations such as WeChat, TikTok, Snapchat, and Twitter [100]. Future research shall also include measures to further describe the use such as motivation and whether they are passive, active social, or active non-social users of each platform. Furthermore, age and cultural sensitivity need to be included, as they influence the choice of platforms that are being used and have an impact on how individuals use different social media platforms [101]. Facebook was categorized as a part of the social networking cluster. Facebook has a separate application for messaging, known as Messenger [102]. We did not include the use of Facebook Messenger to maintain differences from the conversational app WhatsApp, which was part of the study. Although many different things can be performed on the app, it is primarily categorized as a networking app in this study. Future studies shall consider the features that lead people to use each of these platforms excessively.

## 5 Conclusion

With many individuals using social media daily, it is crucial for experts to discover how this new form of communication affects people's everyday lives, relationships, and subjective well-being. The identified clusters can be used to segment social media users concerning their use and PIU, understand them better, and facilitate tailored interventions to help healthier usage. In conclusion, our results should be interpreted with caution as the study was conducted on individuals who wanted to be conscious of their usage. Indeed, the results from our clustering are better applied to those with technology overuse. Our results indicate that PIU may be related to excessive use of a particular Social Media platform. Hence, measuring each social media app usage separately may facilitate better intervention methods to reduce PIU. Those with a high degree of social media usage and at risk of PIU should be encouraged to engage in offline social activities and interactions [103]. Developers of self-regulation and self-monitoring apps to track digital well-being will be able to create strategies that target people with PIU if they understand how to predict behavior and app usage. Personalized and just-in-time interventions tailored to an individual's needs and psychosocial behavior have been effective in treating various behavioral health issues such as obesity, schizophrenia, and smoking [[104], [105], [106], [107]], and can be considered to reduce PIU. Our study can contribute to designing more effective just-in-time adaptive interventions for problematic Internet use.

## Author contribution statement

Khansa Chemnad: Conceived and designed the experiments; Contributed data; Performed the experiments; Analyzed and interpreted the data; Wrote the paper.

Maryam Aziz: Conceived and designed the experiments; Performed the experiments.

Samir Brahim Belhaouari: Conceived and designed the experiments; Contributed analysis tools.

Raian Ali: Conceived and designed the experiments; Contributed data; Analyzed and interpreted the data; Wrote the paper.

## Data availability statement

Data will be made available on request.

## Declaration of interest statement

The authors declare no conflict of interest.

## Funding statement

Open Access funding was provided by the Qatar National Library.

## References

[1] ‘Global Social Media Statistics’ DataReportal – global digital insights. https://datareportal.com/social-media-users.

[2] Auxier B., Anderson M. (2021). https://www.pewresearch.org/internet/2021/04/07/social-media-use-in-2021/.

[3] Sampasa-Kanyinga H., Lewis R.F. (2015). Frequent use of social networking sites is associated with poor psychological functioning among children and adolescents. Cyberpsychol., Behav. Soc. Netw..

[4] Young L., Kolubinski D.C., Frings D. (2020). Attachment style moderates the relationship between social media use and user mental health and wellbeing. Heliyon.

[5] Shoemaker Brino K.A., Derouin A.L., Silva S.G. (Mar. 2022). Problematic internet use in adolescents and implementation of a social media hygiene protocol. J. Pediatr. Nurs..

[6] Klavina A., Veliks V., Zusa A., Porozovs J., Aniscenko A., Bebrisa-Fedotova L. (Sep. 2021). Problematic internet use, related psychosocial behaviors, healthy lifestyle, and subjective health complaints in adolescents. Health Behav. Policy Rev.

[7] Wang W. (2021). Association between problematic internet use and behavioral/emotional problems among Chinese adolescents: the mediating role of sleep disorders. PeerJ.

[8] Abar C.C., Farnett S., Mendola K., Koban K., Sarra S. (May 2018). Relationships between parent–child social media interactions and health behaviors. J. Subst. Use.

[9] Author N. (2016). https://www.pewresearch.org/internet/2016/11/11/social-media-update-2016/.

[10] Boczkowski P.J., Matassi M., Mitchelstein E. (Sep. 2018). How young users deal with multiple platforms: the role of meaning-making in social media repertoires. J. Comput.-Mediat. Commun..

[11] Alutaybi A., McAlaney J., Stefanidis A., Phalp K.T., Ali R. (2018).

[12] Mohammed Abubakar A., Al-zyoud M.F. (2021). Problematic Internet usage and safety behavior: does time autonomy matter?. Telematics Inf..

[13] Beard K.W., Wolf E.M. (Jun. 2001). Modification in the proposed diagnostic criteria for Internet addiction. Cyberpsychology Behav. Impact Internet Multimed. Virtual Real. Behav. Soc..

[14] Griffiths M.D., Kuss D.J., Demetrovics Z., Rosenberg K.P., Feder L.C. (2014). Behavioral Addictions.

[15] Attrill A. (2015).

[16] Chow H. (2017). https://www.semanticscholar.org/paper/Predicting-Problematic-Internet-Use-in-A-Sample-of-Chow/05847e0b0b43c4311a7a87623702ca411c93ace0.

[17] El Asam A., Samara M., Terry P. (Mar. 2019). Problematic internet use and mental health among British children and adolescents. Addict. Behav..

[18] Mohan A., Ravindran S.K. (Apr. 2020). Loneliness and problematic internet use among young adults. Int. J. Cyber Behav. Psychol. Learn. IJCBPL.

[19] Moreno M.A., Jelenchick L.A., Breland D.J. (Aug. 2015). Exploring depression and problematic internet use among college females: a multisite study. Comput. Hum. Behav..

[20] Wright R.R., Evans A., Schaeffer C., Mullins R., Cast L. (2021). Social networking site use: implications for health and wellness. Psi Chi J. Psychol. Res..

[21] McCrory A., Best P., Maddock A. (2020). The relationship between highly visual social media and young people's mental health: a scoping review. Child. Youth Serv. Rev..

[22] Kircaburun K., Alhabash S., Tosuntaş Ş.B., Griffiths M.D. (2020). Uses and gratifications of problematic social media use among university students: a simultaneous examination of the big five of personality traits, social media platforms, and social media use motives. Int. J. Ment. Health Addiction.

[23] Meta. https://about.facebook.com/company-info/.

[24] ‘WhatsApp’, WhatsApp.com. https://www.whatsapp.com/.

[25] Montag C. (2015). Smartphone usage in the 21st century: who is active on WhatsApp?. BMC Res. Notes.

[26] Facebook | Meta. https://about.facebook.com/technologies/facebook-app/.

[27] What is Instagram? | Instagram help centre. https://help.instagram.com/424737657584573.

[28] Statista (2021). https://www.statista.com/statistics/253577/number-of-monthly-active-instagram-users/.

[29] Vaid S.S., Harari G.M. (2021). Who uses what and how often?: personality predictors of multiplatform social media use among young adults. J. Res. Pers..

[30] Sheldon P., Bryant K. (May 2016). Instagram: motives for its use and relationship to narcissism and contextual age. Comput. Hum. Behav..

[31] Rozgonjuk D., Pruunsild P., Jürimäe K., Schwarz R.-J., Aru J. (Sep. 2020). Instagram use frequency is associated with problematic smartphone use, but not with depression and anxiety symptom severity. Mob. Media Commun..

[32] RSPH ‘#Status Of Mind: Social Media And Young People's Mental Health And Wellbeing’, Royal Society Of Public Health And Youth Health Movement, London. https://www.rsph.org.uk/our-work/campaigns/status-of-mind.html.

[33] Verduyn P., Ybarra O., Résibois M., Jonides J., Kross E. (2017). Do social network sites enhance or undermine subjective well-being? A critical review. Soc. Issues Policy Rev..

[34] Rozgonjuk D., Sindermann C., Elhai J.D., Montag C. (Feb. 2021). Comparing smartphone, WhatsApp, Facebook, Instagram, and Snapchat: which platform elicits the greatest use disorder symptoms?. Cyberpsychol., Behav. Soc. Netw..

[35] Błachnio A., Przepiorka A., Senol-Durak E., Durak M., Sherstyuk L. (Mar. 2017). The role of personality traits in Facebook and Internet addictions: a study on Polish, Turkish, and Ukrainian samples. Comput. Hum. Behav..

[36] Balta S., Emirtekin E., Kircaburun K., Griffiths M.D. (2020). Neuroticism, trait fear of missing out, and phubbing: the mediating role of state fear of missing out and problematic Instagram use. Int. J. Ment. Health Addiction.

[37] Apaolaza V., Hartmann P., D'Souza C., Gilsanz A. (Jun. 2019). Mindfulness, compulsive mobile social media use, and derived stress: the mediating roles of self-esteem and social anxiety. Cyberpsychol., Behav. Soc. Netw..

[38] Rozgonjuk D., Sindermann C., Elhai J.D., Christensen A.P., Montag C. (Aug. 2020). Associations between symptoms of problematic smartphone, Facebook, WhatsApp, and Instagram use: an item-level exploratory graph analysis perspective. J. Behav. Addict..

[39] Stănculescu E., Griffiths M.D. (2022). Social media addiction profiles and their antecedents using latent profile analysis: the contribution of social anxiety, gender, and age. Telematics Inf..

[40] Sindermann C., Elhai J.D., Montag C. (2020). Predicting tendencies towards the disordered use of Facebook's social media platforms: on the role of personality, impulsivity, and social anxiety. Psychiatr. Res..

[41] Alutaybi A., Al-Thani D., McAlaney J., Ali R. (Jan. 2020). Combating fear of missing out (FoMO) on social media: the FoMO-R method. Int. J. Environ. Res. Publ. Health.

[42] Przybylski A.K., Murayama K., DeHaan C.R., Gladwell V. (2013). Motivational, emotional, and behavioral correlates of fear of missing out. Comput. Hum. Behav..

[43] Koç H., Şimşir Gökalp Z., Seki T. (2023). The relationships between self-control and distress among the emerging adults: a serial mediating roles of fear of missing out and social media addiction. Emerg. Adulthood.

[44] Rozgonjuk D., Sindermann C., Elhai J.D., Montag C. (2020). Fear of missing out (FoMO) and social media's impact on daily-life and productivity at work: do WhatsApp, Facebook, Instagram, and Snapchat use disorders mediate that association?. Addict. Behav..

[45] Sundar S.S., Limperos A.M. (Oct. 2013). Uses and Grats 2.0: new gratifications for new media. J. Broadcast. Electron. Media.

[46] Kardefelt-Winther D. (Feb. 2014). A conceptual and methodological critique of internet addiction research: towards a model of compensatory internet use. Comput. Hum. Behav..

[47] Brand M. (Sep. 2019). The Interaction of Person-Affect-Cognition-Execution (I-PACE) model for addictive behaviors: update, generalization to addictive behaviors beyond internet-use disorders, and specification of the process character of addictive behaviors. Neurosci. Biobehav. Rev..

[48] Katz E., Blumler J.G., Gurevitch M. (1973). Uses and gratifications research. Publ. Opin. Q..

[49] Kim C., Lee J.K. (Oct. 2016). Social media type matters: investigating the relationship between motivation and online social network heterogeneity. J. Broadcast. Electron. Media.

[50] Long J. (2016). Prevalence and correlates of problematic smartphone use in a large random sample of Chinese undergraduates. BMC Psychiatr..

[51] Dempsey A.E., O'Brien K.D., Tiamiyu M.F., Elhai J.D. (2019). Fear of missing out (FoMO) and rumination mediate relations between social anxiety and problematic Facebook use. Addict. Behav. Rep..

[52] Brand M., Young K.S., Laier C., Wölfling K., Potenza M.N. (Dec. 2016). Integrating psychological and neurobiological considerations regarding the development and maintenance of specific Internet-use disorders: an Interaction of Person-Affect-Cognition-Execution (I-PACE) model. Neurosci. Biobehav. Rev..

[53] Alhabash S., McAlister A.R. (2015). Redefining virality in less broad strokes: predicting viral behavioral intentions from motivations and uses of Facebook and Twitter. New Media Soc..

[54] Peterka-Bonetta J., Sindermann C., Elhai J.D., Montag C. (2021). How objectively measured Twitter and Instagram use relate to self-reported personality and tendencies toward Internet/Smartphone Use Disorder. Hum. Behav. Emerg. Technol..

[55] McAlaney J., Almourad M.B., Powell G., Ali R. (Nov. 2020). Perceptions and misperceptions of smartphone use: applying the social norms approach. Information.

[56] Alshakhsi S., Chemnad K., Almourad M.B., Altuwairiqi M., McAlaney J., Ali R. (2022). Problematic internet usage: the impact of objectively Recorded and categorized usage time, emotional intelligence components and subjective happiness about usage. Heliyon.

[57] Throuvala M.A., Griffiths M.D., Rennoldson M., Kuss D.J. (2019). School-based prevention for adolescent internet addiction: prevention is the key. A systematic literature review. Curr. Neuropharmacol..

[58] Bayer J.B., Triệu P., Ellison N.B. (2020). Social media elements, ecologies, and effects. Annu. Rev. Psychol..

[59] Chen C., Cohen O., Sundar S.S. (2022). Differentiating problematic from habitual Instagram use: a uses and Grats 2.0 perspective. Soc. Media Soc..

[60] Tandoc E.C., Lou C., Min V.L.H. (Jan. 2019). Platform-swinging in a poly-social-media context: how and why users navigate multiple social media platforms. J. Comput.-Mediat. Commun..

[61] Welcome to Python.org’, Python.org. https://www.python.org/.

[62] Wilcockson T.D.W., Ellis D.A., Shaw H. (Jun. 2018). Determining typical smartphone usage: what data do we need?. Cyberpsychol., Behav. Soc. Netw..

[63] Gunduz H.C., Eksioglu S., Tarhan S. (Jul. 2017). Problematic internet usage: personality traits, gender, age and effect of dispositional hope level. Eur. J. Educ. Res..

[64] de-Sola J., Talledo H., de Fonseca F.R., Rubio G. (2017). Prevalence of problematic cell phone use in an adult population in Spain as assessed by the Mobile Phone Problem Use Scale (MPPUS). PLoS One.

[65] Wang H., Zhou X., Lu C., Wu J., Deng X., Hong L. (May 2011). Problematic internet use in high school students in guangdong province, China. PLoS One.

[66] Ko C.-H., Yen C.-F., Yen C.-N., Yen J.-Y., Chen C.-C., Chen S.-H. (Dec. 2005). Screening for internet addiction: an empirical study on cut-off points for the chen internet addiction scale. Kaohsiung J. Med. Sci..

[67] Vigna-Taglianti F., Brambilla R., Priotto B., Angelino R., Cuomo G., Diecidue R. (Nov. 2017). Problematic internet use among high school students: prevalence, associated factors and gender differences. Psychiatr. Res..

[68] Ünal A.T. (Dec. 2020). A comparative study of social media addiction among Turkish and Korean university students. J. Econ. Cult. Soc..

[69] Yudes-Gómez C., Baridon-Chauvie D., González-Cabrera J.-M. (Sep. 2018). Un estudio transcultural Cyberbullying and problematic Internet use in Colombia, Uruguay and Spain: cross-cultural study. Comun. Rev. Científica Comun. Educ..

[70] Demetrovics Z. (2016). Psychometric properties of the problematic internet use Questionnaire short-form (PIUQ-SF-6) in a nationally representative sample of adolescents. PLoS One.

[71] Ursachi G., Horodnic I.A., Zait A. (Jan. 2015). How reliable are measurement scales? External factors with indirect influence on reliability estimators. Procedia Econ. Finance.

[72] Dunn T.J., Baguley T., Brunsden V. (2014). From alpha to omega: a practical solution to the pervasive problem of internal consistency estimation. Br. J. Psychol..

[73] Rehman A.U., Belhaouari S.B. (2022). Divide well to merge better: a novel clustering algorithm. Pattern Recogn..

[74] Moqbel M., Kock N. (Jan. 2018). Unveiling the dark side of social networking sites: personal and work-related consequences of social networking site addiction. Inf. Manag..

[75] Sha P., Sariyska R., Riedl R., Lachmann B., Montag C. (2019). Linking internet communication and smartphone use disorder by taking a closer look at the Facebook and WhatsApp applications. Addict. Behav. Rep..

[76] Al-Kandari Y.Y., Al-Sejari M.M. (2021). Social isolation, social support and their relationship with smartphone addiction. Inf. Commun. Soc..

[77] Faye A.D., Gawande S., Tadke R., Kirpekar V.C., Bhave S.H. (2016). WhatsApp addiction and borderline personality disorder: a new therapeutic challenge. Indian J. Psychiatr..

[78] Faye WhatsApp use and its impact on relationships among doctors: a cross-sectional pilot study. https://www.anip.co.in/article.asp?issn=2588-8358;year=2020;volume=4;issue=1;spage=48;epage=55;aulast=Faye.

[79] Limniou M., Ascroft Y., McLean S. (2021). Differences between Facebook and Instagram usage in regard to problematic use and well-being. *J. Technol. Behav. Sci.*, Oct..

[80] Andreassen C.S., Pallesen S., Griffiths M.D. (Jan. 2017). The relationship between addictive use of social media, narcissism, and self-esteem: findings from a large national survey. Addict. Behav..

[81] Acar A. (May 2008). Antecedents and consequences of online social networking behavior: the case of Facebook. J. Website Promot..

[82] Gerson J., Plagnol A.C., Corr P.J. (Oct. 2017). Passive and active Facebook use measure (PAUM): validation and relationship to the reinforcement sensitivity theory. Pers. Indiv. Differ..

[83] Trifiro B.M., Gerson J. (2019). Social media usage patterns: research note regarding the lack of universal validated measures for active and passive use. Soc. Media Soc..

[84] Ellison N.B., Steinfield C., Lampe C. (2007). The benefits of Facebook “friends:” social capital and college students' use of online social network sites. J. Comput.-Mediat. Commun..

[85] Sherlock M., Wagstaff D.L. (2019). Exploring the relationship between frequency of Instagram use, exposure to idealized images, and psychological well-being in women. Psychol. Pop. Media Cult..

[86] Rahardjo W., Mulyani I. (May 2020). Instagram addiction in teenagers: the role of type D personality, self-esteem, and fear of missing out. Psikohumaniora J. Penelit. Psikol..

[87] Adeyanju G.C. (2021). Behavioural symptoms of mental health disorder such as depression among young people using Instagram: a systematic review. Transl. Med. Commun..

[88] Gao W., Liu Z., Li J. (Dec. 2017). How does social presence influence SNS addiction? A belongingness theory perspective. Comput. Hum. Behav..

[89] Alcaro A., Brennan A., Conversi D. (2021). The seeking drive and its fixation: a neuro-psycho-evolutionary approach to the pathology of addiction. Front. Hum. Neurosci..

[90] Elhai J.D., Tiamiyu M.F., Weeks J.W., Levine J.C., Picard K.J., Hall B.J. (Oct. 2018). Depression and emotion regulation predict objective smartphone use measured over one week. Pers. Indiv. Differ..

[91] Alsayat A., El-Sayed H. (Jun. 2016). 2016 IEEE 14th International Conference on Software Engineering Research, Management and Applications (SERA).

[92] Windgassen S., Moss-Morris R., Goldsmith K., Chalder T. (Mar. 2018). The importance of cluster analysis for enhancing clinical practice: an example from irritable bowel syndrome. J. Ment. Health.

[93] Affouneh S., Mahamid F.A., Berte D.Z., Shaqour A.Z., Shayeb M. (2021). The efficacy of a training program for social skills in reducing addictive Internet behaviors among Palestinian university students. Psicol. Reflexão Crítica.

[94] Koo H.J., Kwon J.-H. (2014). Risk and protective factors of internet addiction: a meta-analysis of empirical studies in korea. Yonsei Med. J..

[95] Khazaei F., Khazaei O., Ghanbari-H B. (Jul. 2017). Positive psychology interventions for internet addiction treatment. Comput. Hum. Behav..

[96] Dutton W.H. (2013).

[97] Koessmeier C., Büttner O.B. (2021). Why are we distracted by social media? Distraction situations and strategies, reasons for distraction, and individual differences. Front. Psychol..

[98] (2020). Expanding Guides On Instagram.

[99] Kuss D.J., Griffiths M.D. (2017). Social networking sites and addiction: ten lessons learned. Int. J. Environ. Res. Publ. Health.

[100] Donnelly E., Kuss D.J. (Nov. 2016). Depression among users of social networking sites (SNSs): the role of SNS addiction and increased usage. J. Addict. Prev. Med..

[101] Alshare K.A., Moqbel M., Merhi M.I. (Jan. 2022). The double-edged sword of social media usage during the COVID-19 pandemic: demographical and cultural analyses. J. Enterprise Inf. Manag..

[102] ‘Messenger’, Facebook. https://www.messenger.com/.

[103] Xiao T. (2021). Effects of basketball and baduanjin exercise interventions on problematic smartphone use and mental health among college students: a randomized controlled trial. Evid. Based Comp. Alter. Med..

[104] Ben-Zeev D., Kaiser S.M., Brenner C.J., Begale M., Duffecy J., Mohr D.C. (2013). Development and usability testing of FOCUS: a smartphone system for self-management of schizophrenia. Psychiatr. Rehabil. J..

[105] Gonul S., Namli T., Huisman S., Laleci Erturkmen G.B., Toroslu I.H., Cosar A. (Mar. 2019). An expandable approach for design and personalization of digital, just-in-time adaptive interventions. J. Am. Med. Inf. Assoc..

[106] Patrick K. (2009). A text message-based intervention for weight loss: randomized controlled trial. J. Med. Internet Res..

[107] Rodgers A. (Aug. 2005). Do u smoke after txt? Results of a randomised trial of smoking cessation using mobile phone text messaging. Tobac. Control.

